# Dentate Granule Cells Are Hyperexcitable in the TgF344-AD Rat Model of Alzheimer's Disease

**DOI:** 10.3389/fnsyn.2022.826601

**Published:** 2022-05-24

**Authors:** Lindsey A. Smith, Anthoni M. Goodman, Lori L. McMahon

**Affiliations:** Cell, Developmental, and Integrative Biology, University of Alabama at Birmingham, Birmingham, AL, United States

**Keywords:** dentate granule cell, hyperexcitability, TgF344-AD rat, Alzheimer's disease, dentate

## Abstract

The dentate gyrus is both a critical gatekeeper for hippocampal signal processing and one of the first brain regions to become dysfunctional in Alzheimer's disease (AD). Accordingly, the appropriate balance of excitation and inhibition through the dentate is a compelling target for mechanistic investigation and therapeutic intervention in early AD. Previously, we reported an increased long-term potentiation (LTP) magnitude at medial perforant path-dentate granule cell (MPP-DGC) synapses in slices from both male and acutely ovariectomized female TgF344-AD rats compared with wild type (Wt) as early as 6 months of age that is accompanied by an increase in steady-state postsynaptic depolarization during the high-frequency stimulation used to induce plasticity. Subsequently, we found that heightened function of β-adrenergic receptors (β-ARs) drives the increase in the LTP magnitude, but the increase in steady-state depolarization was only partially due to β-AR activation. As we previously reported no detectable difference in spine density or presynaptic release probability, we entertained the possibility that DGCs themselves might have modified passive or active membrane properties, which may contribute to the significant increase in charge transfer during high-frequency stimulation. Using brain slice electrophysiology from 6-month-old female rats acutely ovariectomized to eliminate variability due to fluctuating plasma estradiol, we found significant changes in passive membrane properties and active membrane properties leading to increased DGC excitability in TgF344-AD rats. Specifically, TgF344-AD DGCs have an increased input resistance and decreased rheobase, decreased sag, and increased action potential (AP) spike accommodation. Importantly, we found that for the same amount of depolarizing current injection, DGCs from TgF344-AD compared with Wt rats have a larger magnitude voltage response, which was accompanied by a decreased delay to fire the first action potential, indicating TgF344-AD DGCs membranes are more excitable. Taken together, DGCs in TgF344-AD rats are more excitable, which likely contributes to the heightened depolarization during high-frequency synaptic activation.

## Introduction

Increased neuronal activity is directly linked to the production, secretion, and regional deposition of Aβ (Wei et al., 2010; Yamamoto et al., 2015) and tau (Wu et al., 2016; Vogel et al., 2020). While the locus coeruleus (LC) is now known to be the first site of pathological tau accumulation (Braak et al., 2011), cortical regions associated with high metabolic activity, such as the default mode network, including the entorhinal cortex and hippocampus, are early targets of network abnormalities in Alzheimer's disease (AD) (Reiman et al., 2012; Vogel et al., 2020). Functional imaging studies reveal hyperactivity in the hippocampus during mild cognitive impairment (Dickerson et al., 2004) and even in preclinical AD (Quiroz et al., 2010; Reiman et al., 2012). While decreased synaptic inhibition is commonly observed in AD mouse models (Rocher et al., 2008; Verret et al., 2012; Hazra et al., 2013), compensatory remodeling of inhibitory circuits is thought to result from early aberrant excitation (Palop and Mucke, 2016). In support, APP23 × PS45 mice have increased activity of hippocampal CA1 pyramidal neurons prior to plaque formation (Busche and Konnerth, 2016). Additionally, increased intrinsic excitability from mature dentate granule cells (DGCs) is reported in Tg2576 AD mice (Hazra et al., 2013; Nenov et al., 2015).

We previously reported increased postsynaptic depolarization during long-term potentiation (LTP)-inducing high frequency stimulation and enhanced LTP magnitude at medial performant path synapses onto dentate gyrus granule cells (MPP-DGCs) in 6-month-old TgF344-AD rats (Smith and McMahon, 2018; Goodman et al., 2021), a rodent model that more fully recapitulates AD-like pathology in an age-dependent manner (Cohen et al., 2013; Do Carmo and Cuello, 2013; Tsai et al., 2014; Joo et al., 2017; Rorabaugh et al., 2017; Bazzigaluppi et al., 2018; Muñoz-Moreno et al., 2018; Pentkowski et al., 2018; Smith and McMahon, 2018; Voorhees et al., 2018). Specifically, the spatiotemporal spread of synaptic dysfunction in the hippocampus begins in the dentate gyrus (DG) prior to area CA1 in both male and female TgF344-AD rats. At 6 months of age, amyloid plaques are first detected in the hippocampus, and tau tangles are absent, but gliosis is significant (Cohen et al., 2013). In addition, we recently reported a significant loss of noradrenergic (NA) fibers in the hippocampus beginning at 6 months (Goodman et al., 2021) when accumulation of hyperphosphorylated tau (pTau) is present in the LC (Rorabaugh et al., 2017), the origin of hippocampal NA innervation (Loy and Moore, 1979). Furthermore, concurrent with the degeneration of the hippocampal NA input, we observed heightened function of β-adrenergic receptors (β-ARs) at MPP-DGC synapses that were responsible for the enhanced LTP magnitude at 6 months in TgF344-AD rats (Goodman et al., 2021). Importantly, while the β-AR antagonist propranolol prevented the heightened LTP magnitude in TgF344-AD rats, it did not completely abolish the increase in steady-state depolarization during the high-frequency stimulation used to induce LTP (Goodman et al., 2021), suggesting that another mechanism is contributing to the increased postsynaptic depolarization.

Since our previous work detected no differences in spine density or presynaptic release probability (Smith and McMahon, 2018), we considered that heightened intrinsic excitability of DGCs could contribute to the increase in steady-state depolarization at MPP-DGC synapses in TgF344-AD rats during high-frequency stimulation (Smith and McMahon, 2018; Goodman et al., 2021). Thus, we measured passive and active membrane properties using whole-cell current clamp recordings in acute hippocampal slices from female rats to determine if DGCs are hyperexcitable in TgF344-AD rats compared with those of wild type (Wt). While increased excitability in the dentate has been reported in transgenic mice with the same human transgenes (Hazra et al., 2013; Nenov et al., 2015), it is not known whether this functional change also occurs in DGCs in the novel TgF344-AD rat model that more faithfully recapitulates human AD (Cohen et al., 2013; Rorabaugh et al., 2017). Demonstrating that dentate hyperexcitability is a common feature in early disease pathogenesis in both mouse and rat transgenic AD models strengthens the concept that hyperexcitability could be a biomarker for progressing pathology. In this study, we reported decreased rheobase, increased voltage response, increased probability of firing an action potential (AP), decreased sag voltage, and greater spike accommodation in 6-month DGCs in TgF344-AD rats compared with those of Wt. Together, these data show that TgF344-AD DGCs are hyperexcitable, and this gain of function may contribute to the enhanced depolarization during tetanus used to induce LTP we observed at 6 months of age (Smith and McMahon, 2018; Goodman et al., 2021).

## Methods

### Animals

All breeding and experimental procedures were approved by the University of Alabama at Birmingham Institutional Animal Care and Use Committee and follow the guidelines outlined by the National Institutes of Health. TgF344-AD males harboring the amyloid precursor protein Swedish (APP_swe_) and delta exon 9 mutant human presenilin-1 (PS1_Δ*E*9_) transgenes were bred to Wt F344 females (Envigo, Indianapolis, IN), as carried out previously in our lab (Smith and McMahon, 2018). Rats were maintained under standard laboratory conditions (12 h light/dark cycle, lights off at 14:00 h, 22°C, 50% humidity, food (Harlan 2916; Teklad Diets, Madison, WI) and water *ad libitum*). Animals were housed using standard rat cages [7 in. (height) × 144 in^2^ (floor)]. Female TgF344-AD and Wt littermates were aged to 6–8 months for these experiments. Transgene incorporation was verified by polymerase chain reaction (PCR) as described previously (Smith and McMahon, 2018).

### Surgery

Fluctuations in ovarian hormones, particularly 17 estradiol, during the estrus cycle elicit significant effects on hippocampal dendritic spine density, N-methyl-D-aspartate receptor (NMDAR)/α-amino-3-hydroxy-5-methyl-4-isoxazolepropionic acid receptor (AMPAR) ratio, synaptic plasticity, and learning and memory (Woolley and McEwen, 1993; Woolley, 1998; Smith and McMahon, 2005, 2006; Hajszan et al., 2007; Smith et al., 2010; Vedder et al., 2013). Therefore, to eliminate this variable that could confound results, all rats underwent ovariectomy. Briefly, TgF344-AD and Wt littermate female rats were bilaterally ovariectomized (OVX) under 2.5% isoflurane in 100% oxygen, using aseptic conditions. A 10–14 day minimum postoperative interval was used prior to the experimentation, which allows for the depletion of endogenous ovarian hormones as previously published (Smith and McMahon, 2005, 2006; Smith et al., 2010). Importantly, the LTP magnitude at CA3–CA1 synapses in OVX rats 10–14 days post OVX is not different from ovary intact cycling rats at diestrus (when plasma E2 is lowest in ovary intact cycling rats) (Smith et al., 2009). This confirms that 10–14 days of E2 deprivation and the OVX surgery do not negatively affect synaptic function.

### Hippocampal Slice Preparation

Animals were deeply anesthetized *via* inhalation anesthesia using isoflurane, rapidly decapitated, and brains removed. Coronal slices (400 μm) from the dorsal hippocampus were prepared using a Leica VT 1000A vibratome (Leica Microsystems Inc, Buffalo Grove, IL). To preserve neuronal health and limit excitotoxicity, slices were sectioned in low Na^+^ and sucrose-substituted ice-cold artificial cerebrospinal fluid (aCSF) containing [in mM: NaCl 85; KCl 2.5; MgSO_4_ 4; CaCl_2_ 0.5; NaH_2_PO_4_ 1.25; NaHCO_3_ 25; glucose 25; sucrose 75 (saturated with 95% O_2_, 5%CO_2_, pH 7.4)]. Slices were held at room temperature for 1 h in (4-(2-hydroxyethyl)-1-piperazineethanesulfonic acid) (HEPES)-buffered artificial cerebrospinal fluid (aCSF) [in mM: 92.0 NaCl, 2.5 KCl, 2.0 MgSO_4_, 2.0 CaCl_2_, 1.25 NaH_2_PO_4_, 30 NaHCO_3_, 25 glucose, 5 L-ascorbic acid (saturated with 95% O_2_, 5%CO_2_, pH 7.4)] before transfer to the submersion chamber for recordings. HEPES-modified storing ringer was used to reduce cell swelling and slice damage and robustly improves slice health for path clamp recordings in aged animals (Ting et al., 2014). Following the 1 h recovery, slices were transferred to a submersion chamber and continuously perfused (~2–3 ml/min) with standard artificial cerebrospinal fluid (aCSF) [in mM: 119.0 NaCl, 2.5 KCl, 1.3 MgSO_4_, 2.5 CaCl_2_, 1.0 NaH_2_PO_4_, 26.0 NaHCO_3_, 11.0 Glucose (saturated with 95% O_2_, 5%CO_2_, pH 7.4)] held at 26–28°C.

### Whole-Cell Current Clamp

Whole-cell patch clamp recordings were performed from DGCs in the ectal limb (i.e., upper blade). Somas of DGCs were blind patched using borosilicate glass pipettes (3–6 MΩ) filled with intracellular solution containing (in mM) 120 K-gluconate, 0.6 (ethylene glycol-bis(β-aminoethyl ether)-N,N,N′,N′-tetraacetic acid) (EGTA), 5.0 MgCl_2_, 2.0 ATP, 0.3 GTP, 20.0 HEPES, 270–280 mOsm, pH 7.2 adjusted with KOH. To assess intrinsic excitability, somatic current injections were performed in the presence of glutamatergic antagonists, (2R)-amino-5-phosphonovaleric acid (APV, 100 μM), Ro 25-6981 [0.5 μM], and 6,7-dinitroquinoxaline-2,3-dione (DNQX, 10 μM), and the GABAergic antagonist Picrotoxin (100 μM), to block synaptic transmission. Nifedipine [10 μM] was also used to block Ca_V1.1−4_, which contributes to heightened β-AR function in TgF344-AD rats (Goodman et al., 2021). After achieving the whole-cell configuration, cells were voltage clamped at −70 mV for 3–5 min, and the amplifier was then switched to the current clamp. Hyperpolarizing and depolarizing square wave current injections, 800 ms duration at 100 pA increments, were elicited serially from −400 to +900 pA, repeated 3 times, with a 3–5 min rest period between each current injection round and trials averaged within a single cell. Series resistance was monitored at the beginning and the end of each trial, and cells were excluded if there was a ≥20% change. The resting membrane potential (RMP) was obtained at the initial starting potential in the 100 ms prior to the start of the current injection. To avoid contamination by hyperpolarization-activated cation currents (I_h_ or “sag”), which were evident in the first 3 hyperpolarizing current steps (−400 pA, −300 pA, and −200 pA), input resistance (R_I_) was measured at the −100 pA hyperpolarizing current step. Rheobase was measured as the minimum current required to evoke one or more APs during an 800 ms depolarizing current step from RMP; voltage “sag” potential was measured as the difference between the maximum negative peak at −400 pA and when the voltage reaches steady state (i.e., final 160 ms). Membrane time constant, (time for mV to reach 67% of steady state) was calculated at hyperpolarizing steps. The AP number was calculated at each depolarizing current step, and the AP threshold was measured from the first AP fired. AP accommodation was calculated as the instantaneous frequency at each spike interval. AP waveform properties including AP amplitude, half-width, after hyperpolarization (AHP), and rise and decay times were measured. The AP amplitude and AHP were measured relative to the threshold, and half-width was measured at 50% max amplitude. Instantaneous frequency of firing was measured as the frequency between two consecutive spikes and was plotted against spike interval number. This allows for the measurement of burst like firing as well as accommodation. AP interval number was highly variable among cells making interpretation of accommodation at later spike interval numbers difficult. Therefore, to reduce this variability and to ensure similar health of recorded neurons included in the dataset, only cells that spiked more than 5 times at +100 pA were included in the dataset. Delay to the first AP was measured as the time it took from the start of current injection to the peak amplitude of the first AP. Signals were collected using an Axopatch 200B amplifier (Molecular Devices, Sunnyvale, CA). Recordings were low-pass-filtered at 5 kHz, gain 5 × , and sampled at 10 kHz using a Digidata 1440A (Molecular Devices, LLC, San Jose, CA) and stored on a computer equipped with pClamp10 (Molecular Devices, LLC, Sunnyvale, CA).

### Data Analysis

All data were acquired using the electrophysiology data acquisition software pClamp10 (Molecular Devices, LLC, Sunnyvale, CA.) and analyzed using Origin 2016 (OriginLab), Graphpad Prism 7 (GraphPad Software, Inc.), and SPSS 22 (IBM Corp.). *N* (number) is reported as the number of cells recorded from at least 5 animals per genotype with several data sets from 7 rats per genotype (see figure legends). The experimenter was blind to genotype during the experimental procedure and data collection, with genotype only revealed at the final analysis. Results reported at mean ± SEM with significance set at *p* < 0.05 (^*^) determined by unpaired Student's *t*-test assuming (with Welch's correction for unequal variance) or Mann–Whitney *U*-test for non-parametric data. Outliers were determined with a Grubb's test (GraphPad Software, Inc.), and significant outliers were removed.

## Results

### Excitability Is Increased in Dentate Granule Cells From TgF344-AD Rats

**Figure 1:**
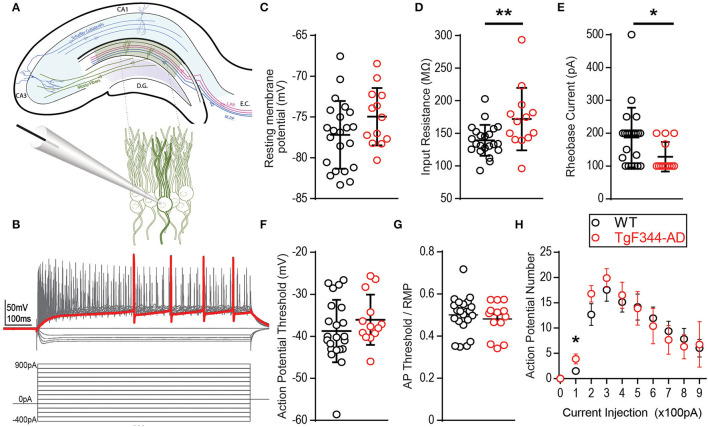
Membrane properties are altered in TgF344-AD dentate granule cells (DGCs). **(A)** Schematic of the trisynaptic circuit in a coronal slice from rat hippocampus (top) with expanded recording setup (bottom). **(B)** Illustration of the experimental protocol: 14 serial 100 pA hyperpolarizing to depolarizing current steps at 800 ms duration from −400 pA to +900 pA. The red trace represents the first depolarizing current step to fire and action potential. **(C)** Mean resting membrane potential is not different between wild type (Wt) (black, 22 cells/8 animals) and TgF344-AD female rats (red, 13 cells/6 animals; *p* > 0.05, but a higher fraction in Wt has values below −80 mV). **(D)** Input resistance is significantly decreased in TgF344-AD DGCs (Wt *n* = 22 cells/8 animals; Tg, *n* = 13 cells/6 animals; **p ≤ 0.01). Data represent mean ± SEM. Significance is determined using the unpaired *t*-test. **(E)** The first depolarizing current step to elicit and action potential, Rheobase (pA), is significantly decreased in TgF344 compared with Wt (*p < 0.05). **(F)** Action potential threshold is not different between genotypes (*p* > 0.05). **(G)** When AP threshold is normalized to RMP, there remains no difference between genotype (*p* > 0.05). **(H)** AP number was enhanced for TgF344-AD rat DGCs at 100 pA current injection (*p* < 0.05) but not at higher current injections. For all panels, Wt, *n* = 22 cells/8 animals; Tg, *n* = 13 cells/6 animals). Data represent mean ± SEM. Significance is determined using the unpaired Student's *t*-test.

Increased DGC excitability has been reported in other transgenic rodent models of AD (Hazra et al., 2013; Nenov et al., 2015; Kim et al., 2021). We recently reported increased steady-state postsynaptic depolarization of DGCs during high-frequency stimulation and heightened LTP magnitude at MPP-DGC synapses in 6-month-old TgF344-AD rats (Smith and McMahon, 2018; Goodman et al., 2021). The increased steady-state depolarization is partially driven by heightened activity of β-ARs at MPP-DGC synapses, as a β-AR antagonist does not completely eliminate the increased depolarization (Goodman et al., 2021). Therefore, we sought to determine if heightened intrinsic excitability could also be occurring and tested this using whole-cell current clamp recordings of DGC neurons. Intrinsic excitability was isolated from synaptic transmission using the AMPAR antagonist DNQX (10 μM), NMDAR antagonist d,l-APV (100 μM), and the GABAergic antagonist picrotoxin (100 μM). Voltage-gated calcium channels Ca_V1.1−4_ were blocked with nifedipine (10 μM) to eliminate any contribution from the heightened β-AR function in TgF344-AD rats (Goodman et al., 2021) (Figure 1A). Incremental hyperpolarizing and depolarizing current steps of 100 pA, 800 ms in duration, were used to assess passive and active membrane properties (for experimental protocol, refer to Figure 1B). RMP was measured immediately after obtaining the whole-cell configuration in the absence of a current injection (I = 0). Although the average RMP recorded from DGCs in TgF344-AD rats was not significantly different from that measured in Wt littermates [Figure 1C; *t*_(28.73)_ = 1.69, *p* = 0.10], Wt DGCs were over 4-fold more likely to rest below −80 mV than their TgF344-AD counterparts (Wt: 7/22 [31.81%] vs. Tg: 1/13 [0.077%]). R_I_ was calculated from the steady-state hyperpolarization generated at −100 pA (in which sag current is undetectable). Input resistance in TgF344-AD DGCs was significantly higher than that measured in Wt littermates [Figure 1D; Tg (*Mdn* = 168.1), Wt (*Mdn* = 133.8), *U* = 69.0, *p* = 0.011]. Together, these data demonstrate modified passive membrane properties in DGCs from TgF344-AD rats.

### DGCs Fire at Lower Depolarizing Current Injections in TgF344 Rats

Fast voltage-gated and slow persistent Na^+^ currents dictate active membrane properties, and alterations in both types of Na^+^ currents have been implicated in AD (Verret et al., 2012; Corbett et al., 2013). To determine if active membrane properties are modified in TgF344-AD rats, we measured AP threshold, rheobase, AP number, and their rise and decay times. Incremental 100 pA depolarizing current steps reliably elicited APs in DGCs from both TgF344-AD rats and Wt littermates (Figure 1). We did not detect a difference in AP threshold between genotypes when measured from baseline [Figure 1F; *t*_(33)_ = 1.11, *p* = 0.27] nor when AP threshold was normalized to the cell's RMP [*t*_(33)_ = 0.65, *p* = 0.52] (Figure 1G). Interestingly, the rheobase, or the minimum depolarizing current needed to generate an AP, was significantly lower in TgF344-AD rats compared with that in Wt DGCs [Figure 1E; *t*_(32.27)_ = 2.542, *p* = 0.016]. This reduced depolarizing current to generate an AP in TgF344-AD rat DGCs is congruent with the increased input resistance in TgF344-AD DGCs (Figure 1D). A decrease in rheobase could lead to an increase in AP number, which was evaluated next at each of the depolarizing current steps. While there was no difference in AP number at current step values higher than 200 pA, TgF344-AD rat DGCs fired more APs at 100 pA [Figure 1H; *t*_(25)_ = 2.26, *p* = 0.033]. The greater number of APs at this low current step suggests an enhanced sensitivity to fire following weak stimulation.

**Figure 2:**
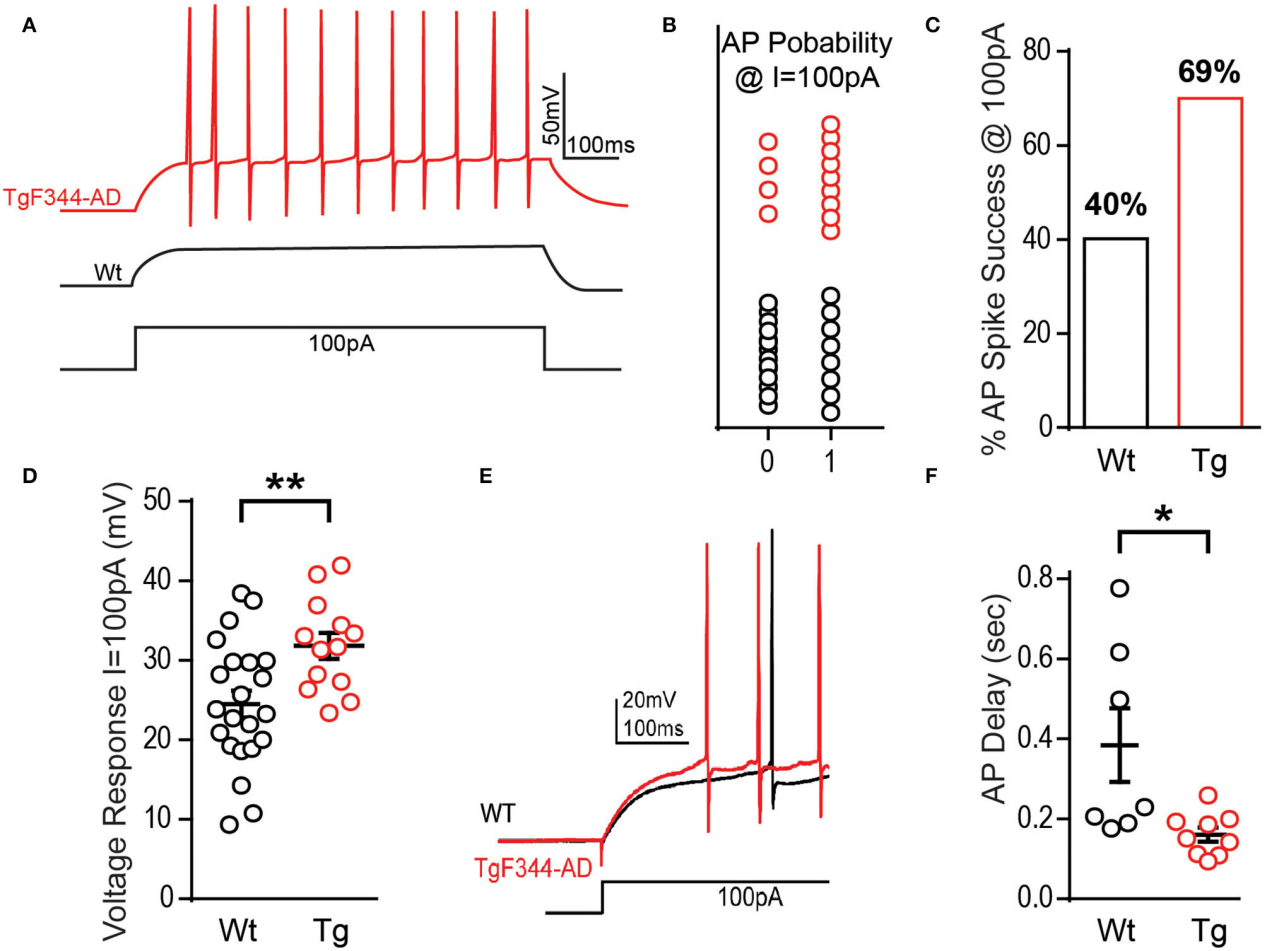
TgF344-AD rats have a lower excitation threshold and fire sooner. **(A)** Example traces from a DGC recorded from a TgF344-AD and Wt littermate to illustrate that +100 pA depolarizing step can elicit action potentials more readily in TgF344-AD rats vs. Wt DGCs. **(B)** The probability of firing an action potential at +100 pA (0 = did not fire an AP, 1 = fired at least one AP) is greater in TgF344-AD DGCs (Wt 9/22 cells fired, Tg 9/13 cells fired). **(C)** DGCs from Wt rats discharged at +100 pA 40.9% of the time compared with a 69.2% success rate observed in TgF344-AD rats. **(D)** Regardless of whether the cell discharged or not, TgF344-AD DGC membranes have a greater voltage change (mV) compared with Wt during the first at +100 pA (Wt, *n* = 22 cells/8 animals; Tg, *n* = 13 cells/6 animals; **p < 0.01). **(E)** Representative traces of the delay to fire the first action potential (Wt = black, Tg = red). **(F)** TgF344-AD DGCs had a decreased delay to fire during the first depolarizing current step at +100 pA (Wt *n* = 7 cells/5 animals, Tg *n* = 9 cells/6 animals; *p < 0.05). Data represent mean ± SEM. Significance is determined using the unpaired Student's *t*-test.

To further investigate initial firing properties, we reasoned that in addition to firing more APs at the minimal depolarizing step as mentioned above, they should also fire more reliably at this minimal depolarizing current injection than Wt DGCs. Indeed, we found that at a depolarizing current injection of +100 pA, the probability of successful AP generation is greater in DGCs from TgF344-AD compared with that in Wt rats, with “0” denoting failure to fire at least one AP and “1” indicating successful AP generation (Figures 2A,B), although no significant difference in the rise time (τ) of the membrane potential resulting from the 100 pA current injection [*t*_(35)_ = 0.017, *p* = 0.986] was detected. In fact, we found that DGCs in TgF344-AD rats were almost 2-fold more likely to fire an AP than DGCs from Wt rats at the smallest depolarizing current injection (Figure 2C). Consistent with this observation, the voltage response during the first depolarizing current step (+100 pA) in TgF344-AD DGCs is significantly increased [Figure 2D; *t*_(31.27)_ = 3.146, *p* = 0.004], and the delay to fire the first AP was significantly reduced [Figures 2E,F; Tg (*Mdn* = 0.15), Wt (*Mdn* = 0.23), *U* = 9.0, *p* = 0.016]. Together, these data show that for the same amount of excitable input, TgF344-AD DGCs fire more consistently and experience a greater steady-state membrane depolarization compared to Wt. This finding is consistent with our previous observations of greater steady-state depolarization as early as 6 months in extracellular dendritic field potential recordings in TgF344-AD dentate following a high-frequency depolarization used to induce LTP (Smith and McMahon, 2018; Goodman et al., 2021).

### Action Potential Kinetics Do Not Account for Increased Excitability in TgF344-AD DGCs

**Figure 3:**
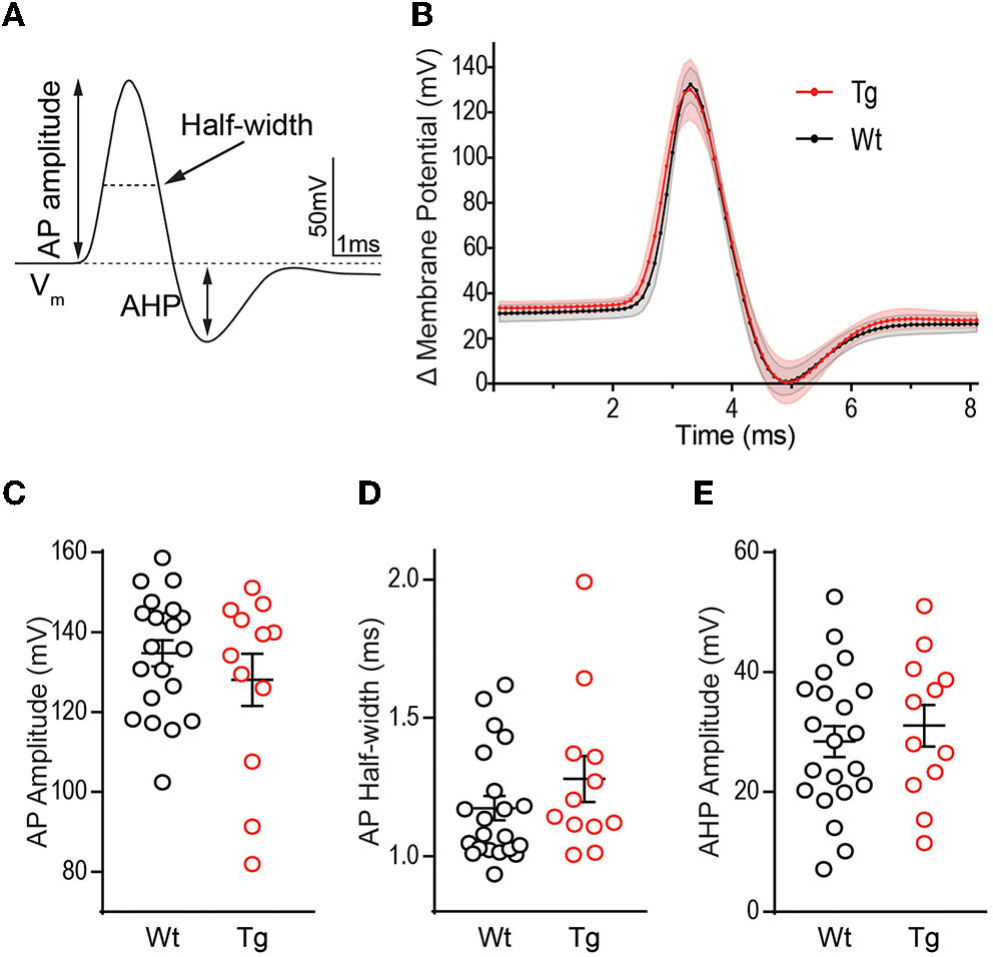
Action potential waveform properties are not different between TgF344-AD and wild-type (Wt) DGCs. **(A)** Schematic of measurements derived from a typical action potential waveform, including, AP amplitude, half-width, and after hyperpolarization (AHP). **(B)** Averages of the first action potential fired at rheobase in each genotype (error bars represent scanning electron microscope (SEM)). **(C)** Action potential height (mV), **(D)** action potential half-width (ms), and **(E)** AHP amplitude (mV) in DGCs from 6 month TgF344-AD and Wt female rats are not different. For all panels, Wt, *n* = 22 cells/8 animals, Tg, *n* = 13 cells/6 animals, *p* > 0.05. Data represent mean ± SEM. Significance is determined using the unpaired Student's *t*-test.

To assess whether changes in rheobase and the increased depolarizing voltage response (Figures 1E, 2D) impact the AP kinetics, we quantified several properties of the AP waveform to include amplitude, rise and decay times, half-width, and the amplitude of AHP (Figure 3A). We chose to quantify kinetics of the first AP fired at rheobase, to prevent modification of AP waveform due to inactivation of Na^+^ and K^+^ channels, as well as activation of currents at hyperpolarized potentials. The first AP waveform was averaged from all cells recorded in each group (Wt = black, Tg = red) (Figure 3B). AP amplitude *t*_(16.52)_ = 0.915, *p* = 0.374 (Figure 3C), half-width *t*_(17.31)_ = 1.123, *p* = 0.277 (Figure 3D), and AHP amplitude *t*_(22.80)_ = 0.615, *p* = 0.272 (Figure 3E) were not significantly different between genotypes. AP rise time and decay were also not different [rise: (*t*_(28)_ = 0.704, *p* = 0.487; decay *t*_(25)_ = 0.220, *p* = 0.828]. Together, these data suggest that voltage-gated Na^+^ and K^+^ channels that mediate membrane depolarization and repolarization are not functionally altered at this early pathological stage in TgF344-AD rats.

### Sag-Mediated Current Is Decreased in TgF344-AD DGCs

**Figure 4:**
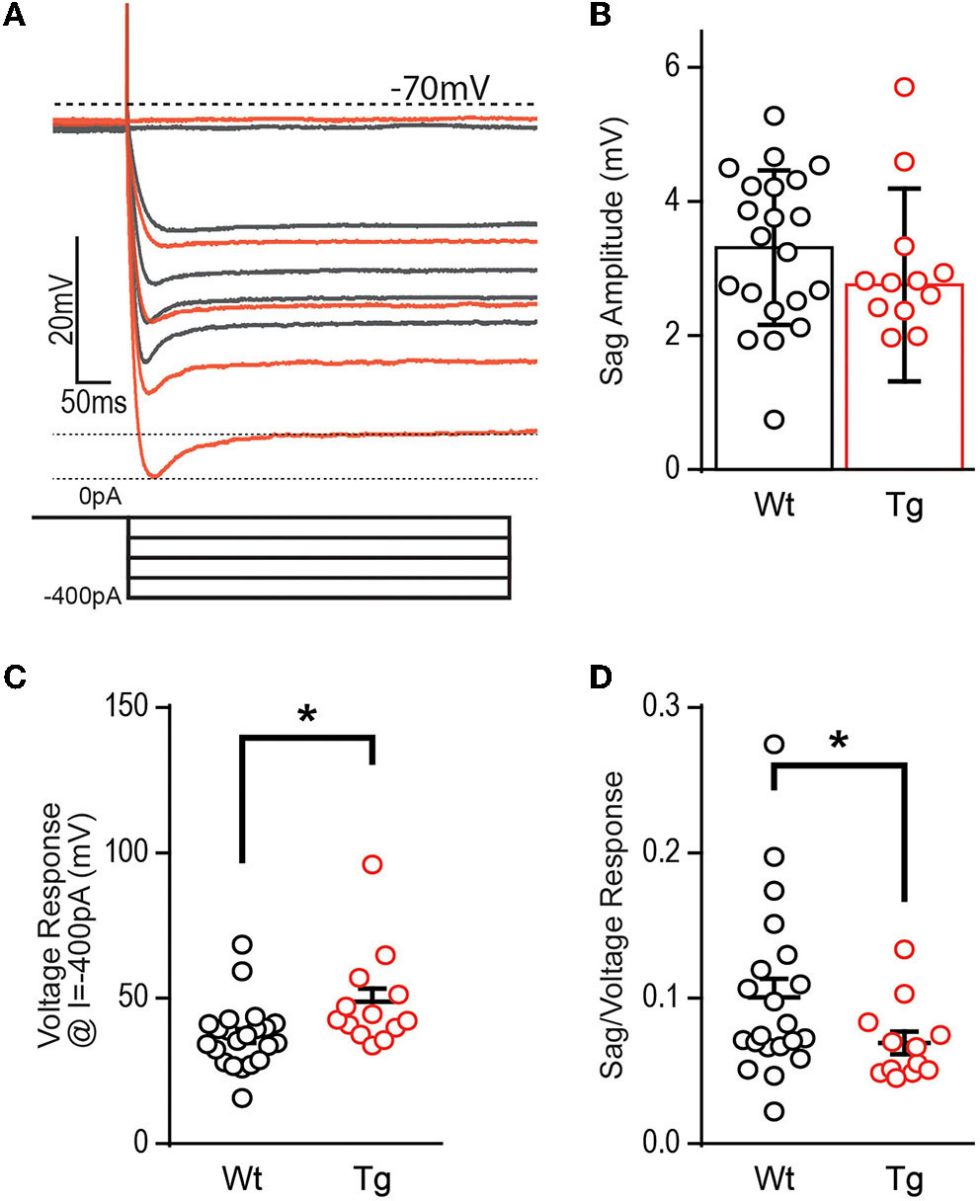
Voltage sag potential is decreased in TgF344-AD DGCs. **(A)** Example trace of “sag” current measurements taken at −400 pA current injection. **(B)** The raw amplitude of the sag current is not different between TgF344-AD and Wt littermate DGCs (Wt, *n* = 21 cells/8 animals; Tg, *n* = 12 cells/6 animals; *p* > 0.05). **(C)** The voltage response at −400 pA is greater in TgF344-AD rats (Wt, *n* = 22 cells/8 animals; Tg, *n* = 13 cells/6 animals; *p < 0.05). **(D)** When sag amplitude is normalized to voltage response a significant deficit in TgF344-AD DGC sag is unmasked (Wt, *n* = 21 cells/8 animals; Tg, *n* = 12 cells/6 animals; *p < 0.05). Data represent mean ± SEM. Significance is determined using the unpaired Student's *t*-test.

Hyperpolarization activates hyperpolarization-activated-cyclic-nucleotide gated currents, I_h_ (HCN channels), which mediate excitability and rhythmic firing in neurons. DGCs typically contain less I_h_ current, although patients with severe epilepsy have increased HCN channel expression confined to the dentate (Poolos et al., 2002; Bender et al., 2005; Poolos and Johnston, 2012), likely a compensatory mechanism in response to overexcitation since HCN channels typically decrease excitability. To determine if decreased HCN channel function could explain the increased excitability observed at this early stage, we additionally measured the “sag,” a voltage signature of HCN channels (Figure 4A). At −400 pA, DGCs in TgF344-AD rats did not show a significant decrease in sag amplitude [Figure 4B; *t*_(21.43)_ = 1.181, *p* = 0.25], but we noted an enhanced voltage response in DGCs from TgF344-AD rats at the −400 pA current injection used to measure sag [Figure 4C; *t*_(18.44)_ = 2.27, *p* = 0.04], which is consistent with the observation that R_I_ is increased in TgF344-AD rats. We detected no change in the membrane time constant in response to hyperpolarizing pulses [(−400 pA) *t*_(34)_ = 0.445, *p* = 0.659; (−300 pA) *t*_(34)_ = 0.966, *p* = 0.341; (−200 pA) *t*_(34)_ = 1.191 48, *p* = 0.242; (−100 pA) *t*_(34)_ = 0.0998, *p* = 0.424]. To compensate for the enhanced R_I_, sag amplitude was normalized to the hyperpolarized voltage response, which resulted in a significant decrease in the sag amplitude in DGCs from TgF344-AD rats [Figure 4D; *t*_(29.94)_ = 2.09, *p* = 0.045]. When taken together, the reduced sag/voltage response may indicate that I_h_ current mediated by HCN channels is impaired in DGCs of TgF344-AD rats.

### TgF344-AD DGCs Have Increased Initial Spike Frequency and Greater Accommodation

**Figure 5:**
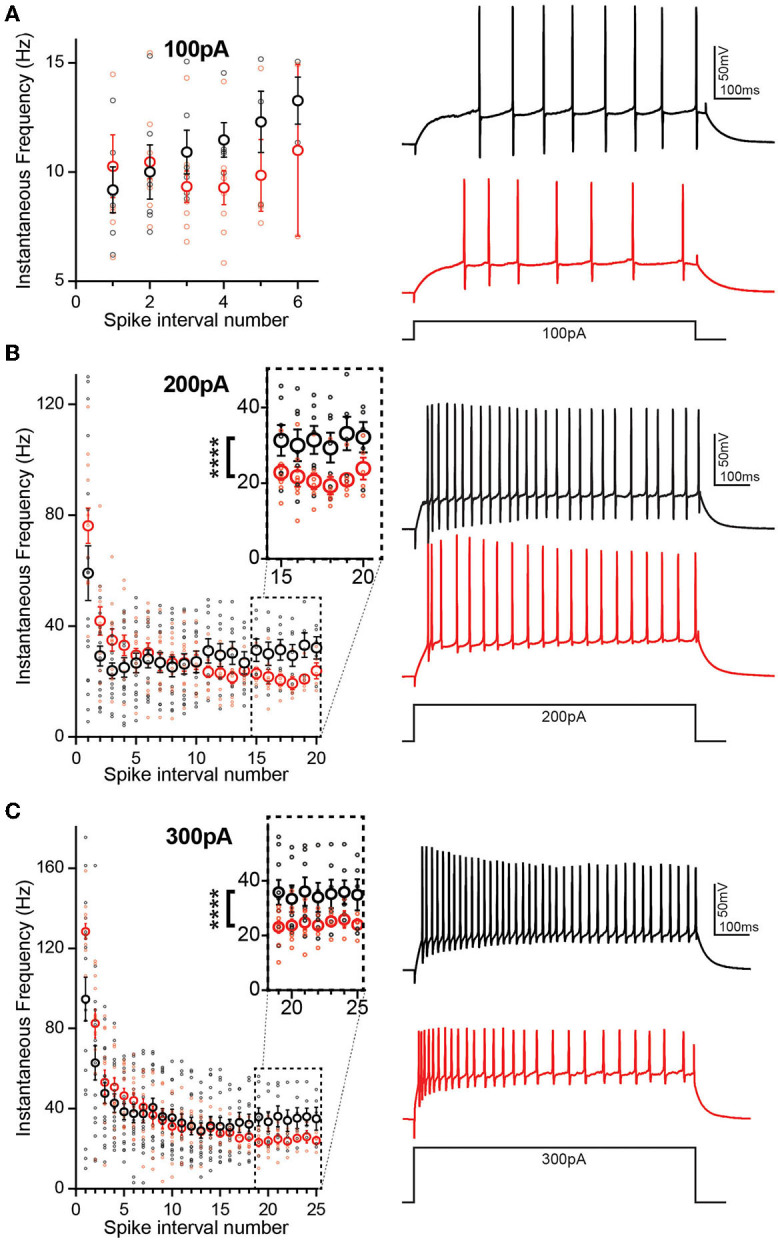
Initial spike frequency and spike accommodation are greater in TgF344-AD DGCs. **(A)** (left) Graph of instantaneous firing frequency (Hz) between action potentials (spike intervals) at 100 pA to include TgF344-AD (red) and Wt (black) DGCs that successfully fired an action potential at +100 pA current injection. (Right) example traces. **(B)** (Left) graph of instantaneous firing frequency (Hz) between action potentials (spike intervals) at 200 pA showing both increased initial spike frequency and greater accommodation (inset) (*p* < 0.0001). (Right) example traces. (Wt, *n* = 7 cells/5 animals; Tg, *n* = 5 cells/5 animals) **(C)** (left) Graph of instantaneous firing frequency (Hz) between action potentials (spike intervals) at 300 pA showing both increased initial spike frequency and greater accommodation (inset) (*p* < 0.0001). (Right) example traces. (****p < 0.0001 Wt, *n* = 7 cells/5 animals; Tg, *n* = 7 cells/5 animals). Data represent mean ± SEM. Significance is determined using the unpaired *t*-test with Welch's correction.

During prolonged depolarization, neurons undergo spike frequency accommodation, where a reduction in the AP frequency occurs over time (Madison and Nicoll, 1984) and is mediated by gradual activation and recruitment of K^+^ channel currents. Enhanced excitability of DGCs in TgF344-AD rats may be a result of increased input resistance and reduced rheobase, yet there is no increase in the AP number above 100 pA current injection suggesting that accommodation may clamp AP number at higher currents. At the first depolarizing step, +100 pA (Figure 5A), differences in instantaneous firing frequency are not interpretable since overall spike number is low and several cells fail to spike. To address this, we assessed accommodation at +200 pA (Figure 5B) when 92% of cells fire an AP as revealed in Brown et al. (2011). We found that DGCs in TgF344-AD rats begin with a mean instantaneous firing frequency of 76.15 ± 6.33 Hz and accommodate to 20.52 ± 2.07 Hz by 17 spike intervals (Figure 5B, red). In contrast, Wt DGCs have a lower initial spike frequency (59.05 ± 9.85 Hz) and accommodate to 31.42 ± 3.71 Hz by steady state (Figure 5B, black). The initial instantaneous firing frequency is not different between TgF344-AD DGCs and Wt (Figure 5B; *p* > 0.05). The second and third intervals, however, suggest a greater instantaneous firing frequency for TgF344-AD DGCs compared with their Wt littermates [*t*_(22.70)_ = 2.032, *p* = 0.054; *t*_(22.68)_ = 2.275, *p* = 0.032, respectively]. When the final 5 spike intervals (15–20) are collapsed (Figure 5B, dashed box), the TgF344-AD rat DGCs have a significantly reduced instantaneous frequency [*t*_(9.66)_ = 11.08, ^****^p < 0.0001], indicating a greater accommodation. Together, these data suggest TgF344-AD DGCs accommodate to a greater degree than Wt, but that the accommodation mechanism is slower to engage. To further validate this finding, a larger depolarizing step (+300 pA) was also used to measure accommodation with similar results as in Brown et al. (2011) (Figure 5C). The first spike interval is significantly shorter for TgF344-AD rat DGCs [*t*_(23.54)_ = 2.906, *p* = 0.008) but quickly becomes comparable to Wt at the second and third interval [*t*_(29.93)_ = 1.758, *p* = 0.089; *t*_(26.31)_ = 0.677, *p* = 0.504, respectively] (Figure 5C). When the final 7 spike intervals (19–25) are collapsed (Figure 5C, inset), the TgF344-AD rat DGCs have a significantly reduced instantaneous frequency [*t*_(11.98)_ = 20.13, ^****^p < 0.0001], again indicating a heightened accommodation. These findings are consistent with the interpretation that the lack of change in total AP number at a given depolarizing current step is a consequence of a simultaneous increase in instantaneous AP frequency and a greater frequency accommodation in DGCs of TgF344-AD rats.

## Discussion

Hyperexcitability in hippocampal circuits is reported in many mouse models of AD (Palop et al., 2007; Roberson et al., 2007; Rocher et al., 2008; Verret et al., 2012; Hazra et al., 2013; Šiškov et al., 2014; Nenov et al., 2015). However, in transgenic rat models of AD, there is a lack of information regarding intrinsic excitability, especially in the DG. In this study, we explored whether intrinsic excitability is altered in the novel TgF344-AD rat model, as it might contribute to synaptic changes such as the increased steady-state depolarization during high-frequency tetanus and the enhanced LTP magnitude we previously reported in TgF344-AD rats (Smith and McMahon, 2018; Goodman et al., 2021), which more faithfully recapitulates human AD pathology than other transgenic rodent models (Cohen et al., 2013; Do Carmo and Cuello, 2013; Rorabaugh et al., 2017).

Passive and active membrane properties of neurons determine RMP, which can be altered by the presence of soluble Aβ (Fernandez-Perez et al., 2016). We did not detect a genotype difference in RMP of DGCs but did find that DGCs in TgF344-AD rats had both a greater likelihood of resting above −80 mV and an elevated R_I_ (Figures 1C,D). A depolarizing shift in RMP has been reported in cortical and CA1 pyramidal cells in various transgenic tau-expressing or AD mouse lines (Minkeviciene et al., 2009; Verret et al., 2012). K^+^ leak and G-protein coupled inwardly rectifying K^+^ (GIRK) channels play a major role in determining RMP (Lüscher and Slesinger, 2010), and their membrane expression could be directly linked to measures of R_I_. Future studies should explore whether changes in these channels underlie the increased R_I_ or even the increased likelihood of a depolarized RMP in the DGCs of TgF344-AD rats.

The enhanced input resistance is further evidenced by the heightened voltage response to both depolarizing and hyperpolarizing currents (Figures 1C, 2D, respectively). Furthermore, the current required to produce APs in TgF344-AD rats is reduced (Figure 1E). Upon closer inspection, the minimal depolarizing current injection used to elicit APs (+100 pA) showed both a significant enhancement in the probability of AP firing, and a decreased delay to fire in DGCs from TgF344-AD rats DGCs (Figure 3). It remains unclear whether these changes are due to altered ion channel expression or function.

K^+^ channels function to dampen membrane excitability and impaired K^+^ channel expression and function is implicated as a mechanism for hyperexcitability in AD models (Scala et al., 2015). Specifically, K^+^ channel dysfunction should reduce the AP width and AHP magnitude (Tamagnini et al., 2015). However, we did not detect a decrease in AP width during the falling phase (Figure 4D), suggesting delayed rectifying K^+^ channels, are intact at this age. The AHP shape is mediated by Ca^2+^-activated K^+^ channels such as the large conductance BK-type channels and the small conductance SK-type channels (Andrade et al., 2012). Unsurprisingly, we did not observe a difference in AHP amplitude in TgF344-AD rats (Figure 3E), suggesting decreased BK and/or SK-type mediated currents likely do not underlie the changes we see. Alternatively, increased intrinsic excitability can be linked to enhanced LTP by an A-type K^+^ channel-mediated increase in back-propagating APs resulting from enhanced dendritic excitability and Ca^2+^ influx (Frick et al., 2004).

Membrane repolarization is mediated in large part by I_A_, which dampens excitatory postsynaptic potentials (EPSPs), raises the threshold for AP initiation, and suppresses back-propagating APs (Hoffman et al., 1997). Activation of I_A_ clamps the membrane below threshold thereby determining the delay to the first AP spike (Storm, 1990). We calculated the time to first spike in DGCs from TgF344-AD rats and Wt littermates and revealed a decreased delay to fire an AP in TgF344-AD rats (Figure 2E), suggesting the effect of I_A_ on dampening excitability may be compromised. While our somatic recordings did not reveal a difference in AHP, whether the threshold for local dendritic I_A_ current is altered or if a downregulation of K^+^ current is responsible for the increased excitability is not known. An elegant study by Kim et al. (2021) in Tg2576 mice shows that decreased expression of Kv4.1 causes DGC hyperexcitability that is associated with impaired pattern recognition. It is possible that loss of Kv4.1 is also occurring in TgF344-AD DG that is leading to increased excitability.

Voltage-gated Na^+^ channels (VGNaCs) are responsible for the initiation of APs and tightly control AP threshold. Decreased VGNaCs have been previously observed in AD mouse models (Verret et al., 2012; Corbett et al., 2013). Specifically, the VGNaC, Na_V1.1_, is decreased in glutamatergic and GABAergic neurons in Tg-AD mouse models leading to increased hyperexcitability, likely through enhanced E/I ratio (Verret et al., 2012; Corbett et al., 2013). We found AP threshold is not different between TgF344-AD and Wt (Figures 1F,G). Furthermore, changes to VGNaC should produce changes to AP amplitude or half-width, yet we did not detect a difference in either measure (Figures 3C,D). Together, these data suggest VGNaC function on DGCs is intact in TgF344-AD rats at 6–8 months, yet whether these channels are decreased on interneurons that innervate DGCs remains to be investigated (Palop et al., 2007; Verret et al., 2012). Unlike the rapidly activating/inactivating VGNaC, persistent Na (I_NaP_) currents do not inactivate, lasting for hundreds of milliseconds with the ability to influence rheobase. These I_NaP_ can augment cell excitability with an additive effect to other depolarizing currents experienced by the cell, can reduce rheobase, and have been implicated in epileptic firing. Our data show a significant decrease in rheobase (Figure 1E), which may indicate a change in I_NaP_.

Hyperpolarization-activated currents can mediate DGC excitability and HCN channel function is impaired in Tg-mouse models of AD (Kaczorowski et al., 2011). While the raw amplitude of the sag current was not different, we found enhanced hyperpolarized shift in the voltage response in TgF344-AD rat DGCs (Figure 4C). Importantly, when the sag amplitude is normalized to voltage response, a deficit in sag response is unmasked, suggesting that for the same amount of membrane hyperpolarization, fewer HCN channels are activated (Figure 4D). These data support that decreased HCN channel function could mediate the DGC hyperexcitability we reported in TgF344-AD rats at 6 months.

Interestingly, while the overall number of APs was not different between genotypes, both the initial AP frequency and spike accommodation were elevated in TgF344-AD DGCs, suggesting the dynamics of AP firing frequency is also altered in TgF344-AD rats. The AP number was highly variable among cells in each group, and therefore, cells that did not meet a minimum spike number (5 spikes at 100 pA, or 17 spikes at 200 pA and above) were excluded for accommodation analysis. Interestingly, when these low-fidelity cells were entered into the analysis, the difference in initial firing frequency was abolished, regardless of spike number, and therefore we cannot rule out the possibility that we were recording from two populations of mature dentate granule cells (Nenov et al., 2015). In fact, variability in the spike number among cells even within the same group indicates there may be multiple populations of mature DGCs from which we are recording (Nenov et al., 2015). While we did not directly measure DGC bursting activity, enhanced instantaneous frequency within the bursting range of 3–8 APs in the TgF344-AD rat may enhance the propagation of signals through the dentate or functionally rearrange feedforward inhibition (Neubrandt et al., 2018). This may be especially true of signals, which otherwise would not evoke a DGC AP due to their increased R_I_. Changes in initial firing frequency and spike rate accommodation support a role for altered Na^+^ and K^+^ channel function in DGCs during short or extended depolarization, and therefore, future studies should aim to determine their functional role in this AD model.

## Conclusion

Excitation inhibition imbalance is an early feature of pathology in the hippocampus of patients with preclinical AD, and this imbalance has been recapitulated in several rodent models of AD-like pathology. We previously reported increased steady-state depolarization and LTP magnitude in TgF344-AD DG at 6 months. While the enhanced LTP was dependent on heightened β-AR function, this enhanced function was not sufficient to account for the heightened SSD (Smith and McMahon, 2018; Goodman et al., 2021). In this study, we used whole-cell current clamp recordings to show that intrinsic excitability is increased in DGCs from TgF344-AD rats, and this provides one mechanistic explanation for heightened excitability in response to high-frequency synaptic activation, which likely contributes to early increase in LTP magnitude.

## Data Availability Statement

The raw data supporting the conclusions of this article will be made available by the authors, without undue reservation.

## Ethics Statement

The animal study was reviewed and approved by University of Alabama at Birmingham Institutional Animal Care and Use Committee.

## Author Contributions

LS and LM designed the study. LS, AG, and LM wrote the manuscript. LS performed the electrophysiological experiments. LS and AG conducted data analysis, statistical tests, and created figures. AG completed the final data analysis and revised the text and figures. All authors contributed to the article and approved the submitted version.

## Funding

This work was funded by grants of NIA R01 AG066489 and NIA R21 AG053067 to LM, F31 AG054087 to LS, and T32-NS-061788 in support of AG.

## Conflict of Interest

The authors declare that the research was conducted in the absence of any commercial or financial relationships that could be construed as a potential conflict of interest.

## Publisher's Note

All claims expressed in this article are solely those of the authors and do not necessarily represent those of their affiliated organizations, or those of the publisher, the editors and the reviewers. Any product that may be evaluated in this article, or claim that may be made by its manufacturer, is not guaranteed or endorsed by the publisher.
